# Post-Translational Modifications of FXR; Implications for Cholestasis and Obesity-Related Disorders

**DOI:** 10.3389/fendo.2021.729828

**Published:** 2021-09-27

**Authors:** Monique D. Appelman, Suzanne W. van der Veen, Saskia W. C. van Mil

**Affiliations:** Center for Molecular Medicine, University Medical Center Utrecht and Utrecht University, Utrecht, Netherlands

**Keywords:** farnesoid X receptor, bile acid signaling, post-translational modifications, SUMOylation, phosphorylation, acetylation, obesity, cholestasis

## Abstract

The Farnesoid X receptor (FXR) is a nuclear receptor which is activated by bile acids. Bile acids function in solubilization of dietary fats and vitamins in the intestine. In addition, bile acids have been increasingly recognized to act as signaling molecules involved in energy metabolism pathways, amongst others *via* activating FXR. Upon activation by bile acids, FXR controls the expression of many genes involved in bile acid, lipid, glucose and amino acid metabolism. An inability to properly use and store energy substrates may predispose to metabolic disorders, such as obesity, diabetes, cholestasis and non-alcoholic fatty liver disease. These diseases arise through a complex interplay between genetics, environment and nutrition. Due to its function in metabolism, FXR is an attractive treatment target for these disorders. The regulation of FXR expression and activity occurs both at the transcriptional and at the post-transcriptional level. It has been shown that FXR can be phosphorylated, SUMOylated and acetylated, amongst other modifications, and that these modifications have functional consequences for DNA and ligand binding, heterodimerization and subcellular localization of FXR. In addition, these post-translational modifications may selectively increase or decrease transcription of certain target genes. In this review, we provide an overview of the posttranslational modifications of FXR and discuss their potential involvement in cholestatic and metabolic disorders.

## Introduction: Farnesoid X Receptor

The Farnesoid X Receptor (FXR) belongs to the family of nuclear receptors, a family of 48 ligand-stimulated transcription factors in humans (1–3). Two FXR genes have been identified: FXRα and FXRβ. FXRβ, encoded by the gene *NR1H5* (Nuclear Receptor Subfamily 1 Group H Member 5), is activated by lanosterol in rodents, rabbits and dogs, but constitutes a pseudogene in humans and primates (4) and is therefore not discussed in this review. The FXRα gene is encoded by the gene *NR1H4* (Nuclear Receptor Subfamily 1 Group H Member 4) and located on chromosome 12q23. FXR has the typical structural organization of members of the nuclear receptor superfamily. It contains an autonomous transactivation domain 1 (AF-1) and an N-terminal DNA binding domain (DBD), which is connected to the ligand binding domain (LBD) *via* a flexible hinge region (5, 6). FXR encompasses 11 exons and is expressed as at least four splice variants (FXRα1-α4) *via* differential promotor usage and alternative splicing (7–9). The four isoforms differ in their AF-1 domain (FXRα1/2 *vs* FXRα3/4) and in a four amino acid insertion (amino acids MYTG) in the hinge region (FXRα1/3 *vs* FXRα2/4) (7, 8). The isoforms are differentially expressed among tissues in humans; for example, FXRα1/2 is highly expressed in the liver, whereas FXR α3/4 are mainly expressed in the intestine (10).

FXR was first described to play a key role in bile acid signaling (1–3). By binding bile acids, FXR regulates transcription of genes involved in bile acid synthesis, transport and their active transport through the enterohepatic circulation (11, 12) (outlined in **Box 1**) and their metabolism by means of stimulation of transcription of glucuronidation and sulphation enzymes. For an overview on the most important FXR target genes in bile acid homeostasis, we refer to **Box 1** (18–20). Briefly, in the enterocyte, FXR activation by postprandial bile acid concentrations initiates the production of fibroblast growth factor 19 (FGF-19), which travels *via* the portal circulation to the hepatocyte, where it induces a signaling cascade inhibiting bile acid synthesis (13–15). In the hepatocyte, FXR activation induces small heterodimer partner 1 (SHP), which in turn also inhibits bile acid synthesis. In addition, FXR regulates the expression of various genes encoding for bile acid transporters, e.g., bile salt export pump (BSEP) (16, 17, 21). Disruption of bile acid flow, also known as cholestasis, due to obstruction of the biliary tract or genetic defects, can result in an accumulation of bile acids in the liver leading to severe hepatocellular toxicity and inflammation (22). Indeed, pharmacological activation of FXR has proven to be beneficial in treating cholestasis (23, 24).

**Box 1:**
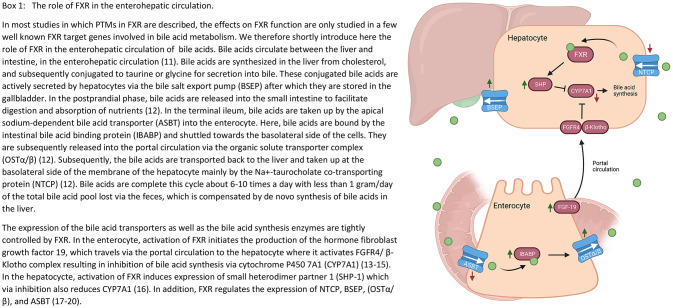
The role of FXR in the enterohepatic circulation. In most studies in which post-translational modifications (PTMs) of the farnesoid X receptor (FXR) are described, the effects on FXR function are only investigated for a few target genes. We therefore shortly introduce here the role of FXR in the enterohepatic circulation of bile acids. Bile acids circulate between the liver and intestine, in the enterohepatic circulation (11). Bile acids are synthesized in the liver from cholesterol, and subsequently conjugated to taurine or glycine for secretion into bile. These conjugated bile acids are actively secreted by hepatocytes via the bile salt export pump (BSEP) after which they are stored in the gallbladder. In the postprandial phase, bile acids are released into the small intestine to facilitate digestion and absorption of nutrients (12). In the terminal ileum, bile acids are taken up by the apical sodium-dependent bile acid transporter (ASBT) into the enterocyte. Here, bile acids are bound by the intestinal bile acid binding protein (IBABP) and shuttled towards the basolateral side of the cells. They are subsequently released into the portal circulation via the organic solute transporter complex (OSTα/β) (12). Subsequently, the bile acids are transported back to the liver and taken up at the basolateral side of the membrane of the hepatocyte mainly by the Na+-taurocholate co-transporting protein (NTCP) (12). Bile acids complete this cycle about 6-10 times a day with less than 1 gram/day of the total bile acid pool lost via the feces, which is compensated by de novo synthesis of bile acids in the liver. The expression of the bile acid transporters as well as the bile acid synthesis enzymes are tightly controlled by FXR. In the enterocyte, activation of FXR initiates the production of the hormone fibroblast growth factor 19 (FGF19), which travels via the portal circulation to the hepatocyte where it activates fibroblast growth factor receptor 4 (FGFR4)/β-klotho complex resulting in inhibition of bile acid synthesis via cytochrome P450 7A1 (CYP7A1) (13–15). In the hepatocyte, activation of FXR induces expression of small heterodimer partner 1 (SHP-1) which via inhibition also reduces CYP7A1 (16). In addition, FXR regulates the expression of NTCP, BSEP, OSTα/β, and ASBT (17–20).

Nowadays, besides its role in bile acid signaling, we recognize FXR as a key regulator of energy metabolism, as FXR is described to be crucial for glucose, lipid and amino acid homeostasis (25–27). FXR is therefore also seen as an attractive treatment target for obesity-related pathology, including insulin resistance and non-alcoholic fatty liver disease (NAFLD) (28–31).

Both conjugated and unconjugated bile acids can activate FXR, in which the most potent activator is the hydrophobic bile acid chenodeoxycholic acid (CDCA, EC_50_ 10 µM). Deoxycholic acid (DCA, EC_50_ 50 µM) and lithocholic acid (LCA, EC_50_ 50 µM) can also activate FXR, however, to a much lesser extent than CDCA, while the hydrophilic bile acid ursodeoxycholic acid (UDCA) and muricholic acid (MCA) cannot activate FXR (2, 32, 33).

The LBD at the C-terminus is implicated in ligand binding, heterodimerization with heterodimer partner Retinoid X Receptor alpha (RXRα) and also required for interactions with transcriptional co-regulators (6, 34, 35). Direct DNA binding of FXR to its response elements (FXREs) occurs *via* the DBD. The consensus FXRE, to which FXR-RXRα complexes bind, consists of an inverted hexamer repeat sequence separated by one nucleotide (referred to as an IR-1) (36, 37), which is situated in promotor and enhancer regions of FXR target genes (18).

Besides classical IR-1 activation, our group recently reported that FXRα2/4, the non-MYTG containing isoforms, additionally activate from an everted hexamer repeat spaced by two nucleotides (referred to as ER-2) (38). These ER-2 sites comprise 90% of all FXR binding positions in the genome, resulting in many additionally regulated genes by FXRα2/4 compared to FXRα1/3. Among others, glucose handling and inhibition of *de novo* lipogenesis are differentially regulated by FXR isoforms (38). Besides transactivation, FXR can also downregulate transcription *via* transrepression, by for example binding to nuclear factor kappa B (NF-κB), thereby prohibiting NF-κB-mediated (26, 39).

The molecular basis by which FXR activity is modulated in health and disease states still remains largely unexplored. Recently, multiple studies have focused on the post-translational modifications (PTMs) of FXR (40–45). These modifications can occur in response to nutrients and other external factors, but can also be regulated *via* inflammation-induced transcription. In this review, we provide an overview of the PTMs which regulate FXR expression and/or function and discuss how these modifications may impact and/or contribute to the disease phenotypes in cholestasis and obesity-related disorders.

## FXR Post-Translational Modifications

### Phosphorylation

Phosphorylation is a modification in which a phosphate group is added to a serine, threonine or tyrosine by a kinase. This reversible modification can regulate different intracellular processes, e.g. protein activity, localization and stability (46).

FXR can be phosphorylated in the AF-1 domain at Y67 upon feeding (47). This phosphorylation was not observed in fibroblast growth factor 15 (FGF-15, FGF-19 in humans) knockout mice, suggesting that FXR-Y67 phosphorylation may be FGF-15 dependent. The authors claimed that meal consumption in these FGF-15^-/-^ mice, did not lead to FXR nuclear localization, which they did observe in wild type (WT) mice (47), thereby suggesting that FGF-15/19 regulates FXR nuclear localization *via* Y67 phosphorylation. In line with this, they demonstrated that FGF-19 treatment of primary hepatocytes led to a shift from cytoplasmic to nuclear translocation of WT FXR, but not of the phospho-defective FXR-Y67F mutant. Concomitant to this, in FXR-Y67F expressing hepatocytes, a decreased interaction with RXR was observed, as well as a decrease in FXR target gene expression. In line with this, the opposite effect was observed in FGF-19 treatment in WT mice (47). Src (proto-oncogene tyrosine-protein kinase Src) and SHP2 (Src homology region 2 domain-containing phosphatase-2) were shown to mediate the FGF-15/19-stmulated phosphorylation of FXR at this Y67 position. In conclusion, FGF-19 activates the FGFR4 (fibroblast growth factor receptor 4) - Src cascade, resulting in FXR nuclear localization, and thereby its availability to interact with RXR and thus in enhanced target gene activation (47).

Several studies have shown that bile acids can activate protein kinase C (PKC) in hepatocytes (48–50). More specifically, UDCA, used for the treatment of cholestatic liver disease, stimulates hepatobiliary excretion of bile acids through PKCα activation (51). Pre-treatment with a PKC inhibitor abolished the increase in CDCA-induced SHP expression, suggestive of PKC regulating FXR activity. Indeed, activation of PKC not only strongly enhanced FXR activity 80-fold, but also increased FXR phosphorylation based on two-dimensional gel analysis (40). To identify the responsible PKC, *in vitro* phosphorylation studies were performed revealing that an FXR protein fragment (106-196 amino acid), was preferentially phosphorylated by PKCα and β1 (40). This fragment contains three potential phosphorylation sites S117, S135 and S154. Replacement of the serine with alanine at S135 or at S154 significantly decreased phosphorylation of FXR while S117A had no effect on FXR phosphorylation (40). This demonstrates that FXR can be phosphorylated at S135 and S154 by PKCα. S135 and S154 single mutants partially impair the PKC activated-CDCA induced FXR activity, while the double mutant completely prevented the enhancement of CDCA-induction by PKC activation on FXR activity (40). The double mutant decreased transcriptional activity with 80%, thereby suggesting that phosphorylation of either S135 and/or S154 promotes its transcriptional activity. These data are difficult to rhyme with data from Hashiguchi et al, who described that S154 phosphorylation decreased FXR activity and promotes its degradation in COS-1 cells (52). Further studies need to address the effects of S154 phosphorylation on FXR function and expression more precisely, but this discrepancy may be due to the different model systems used and the cofactors expressed in these cell lines (HepG2 cells *versus* COS-1 cells).

Besides FXR phosphorylation within the AF-1 and DBD domain FXR can also be phosphorylated within its hinge domain. This is mediated by AMPK (AMP-activated protein kinase A) (53), a kinase which plays a key role in maintaining energy homeostasis (54). AMPK was shown to interact with the hinge domain of FXR in HepG2 cells. AMPK activation by AICAR/metformin, resulted in FXR phosphorylation, and a decrease in FXR target gene expression in multiple mouse and human cell lines. This suggests that phosphorylation of the FXR hinge domain is negatively affecting its function. Mutating the *in silico* predicted phosphorylation site S250 to alanine, abolished the repressive effect of AMPK activation on FXR target gene expression. Also in mice, metformin treatment repressed the taurocholic acid (TCA)-induced FXR target gene expression, although by itself metformin had no effect on FXR target gene expression (53).

Lastly, FXR phosphorylation also occurs in the LBD. It was shown that specific inhibitors of PKCζ reduced BSEP promoter activity which was confirmed by PKCζ knockdown (55). PKCζ can be directly activated by phosphatidylserine (PS), which is located within the plasma membrane (56). In the membrane, the phospholipid concentration is tightly regulated by ATP8B1 (ATPase phospholipid transporting 8B1), which flips PS from the outer to the inner leaflet of the membrane (57). Interestingly, knockdown of ATP8B1 resulted in diminished FXR localization within the nucleus, which was also observed in PKCζ knockdown in ATP8B1 expressing UPS cells (55). Phosphorylation prediction tools predicted T442 as the potential phosphorylation site in the FXR LBD. The phospho-defective mutant, T442A, decreased nuclear localization of FXR, while the phospho-mimetic FXR mutant (FXR-T442E) showed increased nuclear localization (55). This suggests that ATP8B1, by regulating PS ratios in the membrane, activates PKCζ which in turn phosphorylates FXR at T442 resulting in increased BSEP activity and increased bile acid transport into the bile canaliculi. Lastly, S327 is identified as the phosphorylation site involved in the SUMOylation of FXR, which is explained in more detail in the next section.

Altogether, FXR phosphorylation is a frequent modification that occurs at least on 6 different sites in multiple FXR domains and by multiple kinases.

### SUMOylation

The small ubiquitin-like modifier (SUMO) family is present in all eukaryotes which is highly conserved across species. Currently, at least five SUMO paralogs have been reported: SUMO1 to SUMO-5, which differ in their cellular distribution and function (58, 59). SUMOylation is mostly associated with the regulation of protein-protein interactions, mediating the localization and function of target proteins, including nuclear receptors (60). The SUMO group is attached to its target residue *via* a two or three-step process in which the last enzyme, an E3 SUMO ligase, determines target protein specificity (61–63).

To answer the question whether FXR could potentially be SUMOylated, FXR interaction with E2 and E3 ligases was investigated. The E2 ligase UBE2I (ubiquitin conjugating enzyme E2 I) and E3 ligase PIASγ (protein inhibitor of activated STAT) were shown to interact with FXR (42).

Next, the targeted SUMO-binding residue within FXR was investigated. SUMO binds to lysine residues preferably within the minimal consensus motif ψKxE/D, where “ψ” represents a hydrophobic amino acid and “x” refers to any amino acid (64). This motif is predicted twice in human FXR and the lysine residues within this minimal consensus motifs are located at K277 and K122 (65). SUMO2 interaction with FXR occurs at K277, but not at K122, as shown by IP in which FXR-K277R reduced SUMO2 binding to FXR, while K122R had no effect on this interaction (42). Additionally, the FXR-K277R mutant mice had a substantially decreased SUMO2-mediated SUMOylated fraction of FXR in the liver, which did not affect the expression of the FXR target genes BSEP and SHP (42). SUMO2 binding to FXR is mediated by PIASγ, as overexpression of PIASγ substantially increased SUMO2-FXR levels (42, 66). Accordingly, siRNA mediated knockdown of PIASγ almost fully abolished SUMO2 modification of FXR (42). SUMO1 modified FXR levels were only partially reduced in both K277R and the K122R transfected COS-1 cells, suggesting that FXR is SUMO1 modified also at other sites (42, 43). It is not yet elucidated whether PIASγ or another E3 ligase is responsible for SUMO1 interaction with FXR. Notably, mutating both K122 and K277 did not result in a completely abolished FXR-SUMO2 and FXR-SUMO1 conjugation, implicating the existence of other SUMO-modified sites within FXR (42, 67).

Further investigation demonstrated that FXR contains a non-consensus SUMOylation site (pSUM motif) at residue K325, located in LBD (68). This pSUM motif consists of the sequence ψKxS, which becomes fully competent in SUMO conjugating upon phosphorylation of the serine residue, thus providing the necessary negative charge for SUMOylation. The serine within this motif, S327 is part of a canonical SxxE/D consensus site for casein kinase 2 (CK2) and due to this, likely responsible for the phosphorylation of FXR-S327. Indeed, inhibition of CK2 severely impaired FXR-SUMO2 conjugation, while expressing of the catalytic CK2α1 subunit increases FXR-SUMOylation (69). Mimicking the consensus SUMOylation motif at residue 327, by replacing the serine with glutamic acid (S327E), thereby providing the negative charge of this residue, led to an increase in SUMO2 conjugation levels (69). This demonstrates that the phosphorylation of S327 is required for SUMO2 modification of FXR-K325. In addition, K325R mutation severely impaired the effect of UBE2I and PIASγ on SUMO2 conjugation (69).

SUMO1 binding to FXR inhibits FXR-RXRα transactivation from BSEP and SHP promotors (43). In line with this, knockdown of SUMO1 resulted in increased BSEP and SHP promoter-luciferase activity (43). SUMO2 binding to FXR-K277 inhibited expression of NF-κB target inflammatory genes without affecting FXR-RXRα-mediated FXR target genes BSEP and SHP (42). An inverse relation with inflammation has also been observed, in which FXR SUMOylation decreased rapidly upon LPS (lipopolysaccharide) treatment. In addition, this LPS-mediated reduction could be inhibited by treatment with the FXR agonist obeticholic acid (OCA) (67). In this context, the SUMO paralog was not identified. The modification of K327 led to an increase in FXR activity, as demonstrated by a SHP reporter assay, but also to FXR degradation as will be further explained in the ubiquitination section (69).

Thus, FXR SUMOylation can occur at multiple sites, for which the E3 ligase PIASγ is required. Depending on the SUMO site and SUMO paralog, SUMOylation of FXR either downregulates transcriptional activity on its targets BSEP/SHP, inhibits expression of NF-κB targets inflammatory genes or is targeted for degradation.

### Acetylation

The most common form of protein acetylation is the acetylation of lysine residues (70). This results in disruption of salt bridges and introduces steric bulk that can alter protein-protein interactions, protein-DNA interactions and stability (71–73). To answer the question whether FXR could potentially be acetylated, interaction of FXR with P300, a histone acetyl transferase that catalyzes the acetylation of lysine residues, was investigated (74). P300 was shown to interact with FLAG-tagged FXR, overexpressed in mouse liver, suggestive of FXR acetylation (74). The level of FXR acetylation is likely to be balanced between acetylation and deacetylation of the protein. The Sirtuin 1 (SIRT1) deacetylase has been shown to work together with P300 (75). Indeed, also an interaction between and FXR and SIRT1 interaction was demonstrated in mouse liver (41). In these mice, CA feeding reduced the interaction between SIRT1 and FXR, which is in line with the increased interaction with P300 (41).

Subsequently, acetylated lysines within FXR were identified by tandem mass spectrometry. This approach demonstrated that both K157 and K217 were acetylated (41). Pulldown of an acetylation defective mutant (K157R or K217R) demonstrated that K157R hardly affected overall FXR acetylation, while K217R reduced the overall FXR acetylation significantly. Mutating both sites nearly abolished FXR acetylation, thereby demonstrating that probably these are the only two acetylation sites within FXR, of which K217 is the predominant acetylation site. K217R resulted in decreased protein stability on the one hand, but also elevated activation from a FXRE/SHP promoter, which is presumably caused by stabilization of the RXRα-FXR heterodimer. The opposite effect was observed in the acetylation-mimicking mutant (K217Q) (41). The acetylation defective FXR double mutant (K157R/K217R) increased FXR target gene expression, but also led to decreased FXR expression, which could be prevented by blocking the proteasome. These results suggest that FXR is a target of ubiquitin-proteasomal degradation, and acetylation of FXR increases protein stability by inhibiting its degradation.

As expected, SIRT1-FXR interaction resulted in reduced FXR stability, increased FXR-RXRα heterodimerization and hence increased transcriptional activity. In liver specific SIRT1^-/-^ mice, FXR acetylation was elevated in line with reduced FXR target gene expression, while overexpression of SIRT1 resulted in a decreased acetylation and an increased FXR-RXRα heterodimerization and stronger binding to the SHP promoter, which could indicate enhanced FXR target expression (76). In line with this, downregulation of SIRT1 led to decreased BSEP expression (76).

In conclusion, FXR is acetylated by P300 at K157 and K217 and deacetylated by SIRT1. FXR acetylation increases its stability, but reduces FXR-RXRα heterodimerization leading to a reduction in FXR target gene expression.

### Ubiquitination

Ubiquitination is the attachment of a small (76 amino acid) protein to an acceptor lysine in a target protein (77), which requires a three-step enzymatic reaction involving E1, E2 and E3 ligases and can be reversed by de-ubiquitination enzymes (78). FXR overexpression in HEK293 cells resulted in a reduction in FXR ubiquitination levels in GW4064-stimulated compared to vehicle control, suggesting that activation of FXR is linked to its de-ubiquitination (69).

Ubiquitination of FXR was shown to be regulated by SUMOylation of FXR-K325 by SUMO2, which in turn is dependent on CK2-mediated phosphorylation of S327. SUMOylated FXR-K327 is recognized by RING-domain-containing ubiquitin E3 ligase (RNF4), a poly-SUMO-specific E3 ubiquitin ligase, involved in degrading specific target proteins by poly-ubiquitination. Indeed, WT RNF4 overexpression induced FXR degradation, while mutant RNF4 resulted in reduced FXR ubiquitination and concomitantly increased FXR activity, as demonstrated in HEK293 and HepG2 cells (69). Interestingly, SUMOylation of FXR-S327 is not the only trigger to induce FXR ubiquitination and degradation. Phosphorylation of FXR-S154 also leads to degradation of FXR as demonstrated by poor expression of the phospho-mimetic FXR-S154D mutant which could be restored by blocking the proteasome in COS-1 cells (52). Proteomic analyses in mouse liver revealed an ubiquitination site at K370 within FXR (79). As no further studies were undertaken, confirmation is still awaited on whether there is a causal relation with FXR degradation for this ubiquitination site. Of note, this specific lysine residue in not present in human FXR. In conclusion, human FXR can be ubiquitinated in response to either SUMOylation of FXR-K325 or FXR-S154 phosphorylation.

### Glycosylation

Berrabah et al. demonstrated that FXR can be modified through O-GlcNAcylation in response to high glucose levels (45). O-linked GlcNAcylation is more abundant on nuclear proteins compared to cytosolic proteins and involves the addition of B-D-N-acetylglucosamine by O-GlcNAc transferase (OGT) to a Serine (S) or Threonine (T) residue, often located within a proline-valine-S/T motif. This PTM is reversible and cycles on proteins with a timescale similar to protein phosphorylation (80, 81). FXR contains a proline-valine motif prior its threonine residue within its AF-1-domain, pointing towards a possible O-GlcNAc modification at this location (82). FXR was shown to interact with OGT and with meningioma expressed antigen 5 (MGEA5), which function in attaching and removing GlcNAc, respectively (45). FXR O-GlcNAcylation was shown to enhance FXR protein stability and induce BSEP and SHP expression. Mutation of the conserved serine 62 to an alanine (S62A) within the proline-valine-S/T motif, reduced FXR O-GlcNAcylation in line with a 70% reduction in FXR transcriptional activity (45), thereby showing that S62 O-GlcNAcylation is required for optimal FXR transcriptional activity. The increase in FXR O-GlcNAcylation induces BSEP expression in WT HepG2 cells, however, no mutation analysis was performed to validate this further (45). Furthermore, the modification still occurs in the S62A mutant suggesting that other sites are possibly also targeted for O-GlcNAcylation. In conclusion, FXR O-GlcNAcylation at S62 and potentially other sites, is mediated by OGT and MGEA5 expression and results in increased FXR activity.

### Methylation

Methylation involves the transfer of a methyl group to an arginine or lysine residue within a protein (83). A well-conserved consensus recognition site for Set7/9 lysine methylation was predicted in the hinge region of FXR, (KSKR, amino acid 204-207) (84), which triggered Balasubramaniyan et al. to investigate whether FXR is indeed methylated (44). Depletion of Set7/9, a lysine methyltransferase, in Huh7 cells resulted in a significant decrease in the FXR target genes SHP and BSEP, demonstrating that Set7/9 influences FXR function possibly by methylation (44, 85, 86). Excitingly, mutating lysine 206 to an arginine (K206R) completely abrogated the methylation of FXR by Set 7/9, in line with reduced BSEP expression (44). In addition, specific binding of FXR/RXRα to the FXRE was markedly enhanced by overexpression of Set7/9, while this was reduced in the FXR-K206R mutant. Accordingly, knockdown of Set7/9 resulted in a decreased occupancy of FXR at the SHP and BSEP promoters, as shown in chromatine immunoprecipitation (ChIP) assays. This suggest that FXR-K206 methylation stabilizes FXR and, possibly by promoting its interaction with RXRα, leads to enhanced transcriptional activity of FXR.

## (Potential) Impact of FXR PTMs in Disease

**Figure 1:**
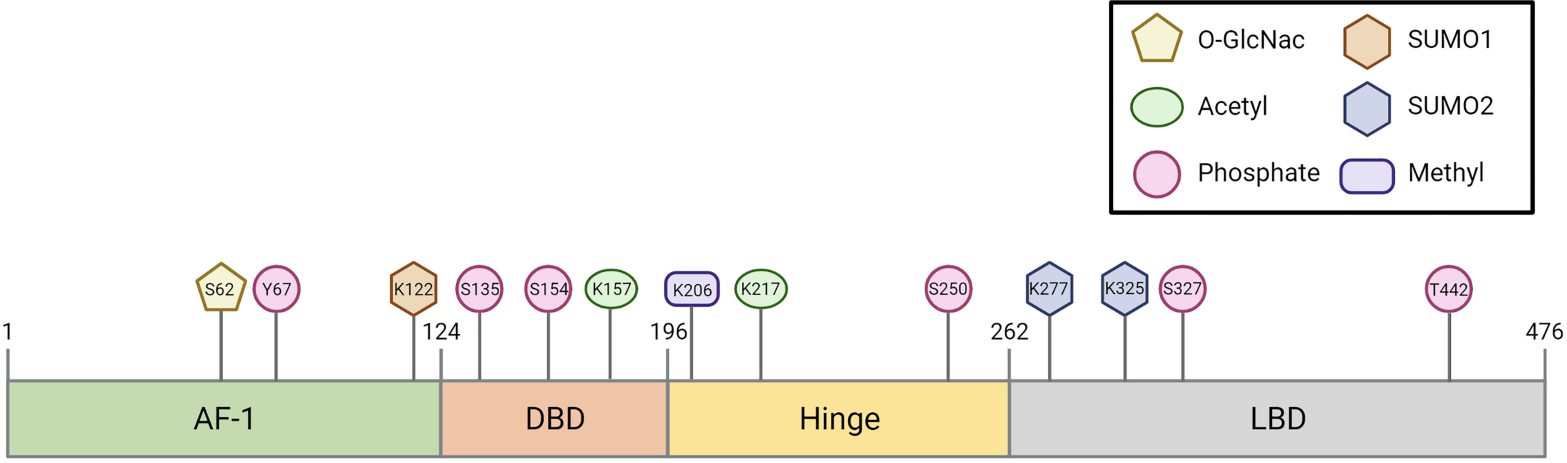
Schematic representation of the post-translational modifications within the FXR protein.

In the first part of this review, we discussed all known PTMs that can occur on FXR and the molecular consequences (**Figure 1** and **Table 1**). In the following chapters, we will discuss the potential involvement of PTMs in cholestasis and obesity-related disorders.

**Table 1:** FXR modifications and their effects on FXR function.

Amino acid	Modification	Enzyme mediating the modification	Enzyme mediating the removal of the modification	Effect modification on FXR:					Ref
				Stability	Expression	Nuclear localization	Function	FXR-RXRα interaction	
S62	O-glcNACylation	OGT		↑	=	=	↑	–	(45)
Y67	Phosphorylation	Src		–	–	↑	↑	↑	(47)
K122	SUMOylation							↓	(43)
S135	Phosphorylation	PKC		–	–	=	↑	=	(40)
S154	Phosphorylation	PKC		–	↓	=	↑	=	(40, 52)
K157	Acetylation	P300	SIRT1	–	–	–	↓	–	(41, 74)
K206	Methylation	Set7/9		–	–	–	↑	↑	(44)
K217	Acetylation	P300	SIRT1	↑	–	–	↓	↑	(41, 74)
S250	Phosphorylation	AMPK		–	–	↓	↓	–	(53)
K277	SUMOylation	PIASγ		–	–	–	=	=	(42)
K325	SUMOylation	PIASγ		–	–	–	↑	↓	(69)
S327	Phosphorylation	CK2		↓	↓	–	–	–	(69)
T442	Phosphorylation	PKCζ		–	–	↑	↑	–	(55)
	Ubiquitination	RNF4		–	↓	–	↓	–	(69)

The arrows indicate positive or negative effect; (=) not affected by this modification (–); not investigated.

### Cholestasis

Cholestasis is characterized by impairment of bile formation and/or bile flow due to (genetic) defects in bile acid formation, bile acid excretion or mechanical obstructions in bile flow. As a consequence of impaired bile flow, (toxic) hydrophobic bile acids accumulate within the hepatocyte, thereby causing damage, driving inflammatory reactions and disrupting cell membrane integrity (22). Intrahepatic activation of FXR by the increased bile acid concentration is expected to reduce the bile acid load in hepatocytes by limiting expression of bile acid uptake transporters, reducing bile acid synthesis and stimulating bile acid excretion. However, these intrinsic protective mechanisms are not sufficient to prevent cholestatic liver injury (23, 24). Pharmacological activation of FXR with synthetic agonists is thought to reduce bile acid synthesis and hepatic bile acid load to a greater extent, thereby successfully reducing cholestasis-induced liver injury (23, 24).

During obstructive cholestasis, it has been shown that SUMOylation pathways were significantly altered. ChIP analysis of mouse liver after 3 days of bile duct ligation (BDL) showed a marked increase in recruitment of SUMO1, SUMO2, UBE2I, and PIAS1 to the promoters of BSEP and SHP compared with sham-operated mice (43). FXR recruitment to the BSEP/SHP FXRE was not changed upon BDL (43). The increase in SUMO1/2 recruitment implied that the SUMOylation is independent of FXR binding to the FXRE. Unfortunately, the effect on BSEP and SHP expression was not investigated, nor the SUMO paralog mediating this SUMOylation, or the effect of FXR SUMOylation on its function. At this point, we therefore are not sure whether FXR SUMOylation would be contributing or ameliorating the cholestatic phenotype, as both effects of FXR SUMOylation have been described (see **Table 1**). Interestingly, co-incubation of OCA with general SUMOylation inhibitors drastically decreased fibrosis in bile duct ligated mice, as demonstrated by a reduction in hepatic stellate cell activation (87).

**Figure 2:**
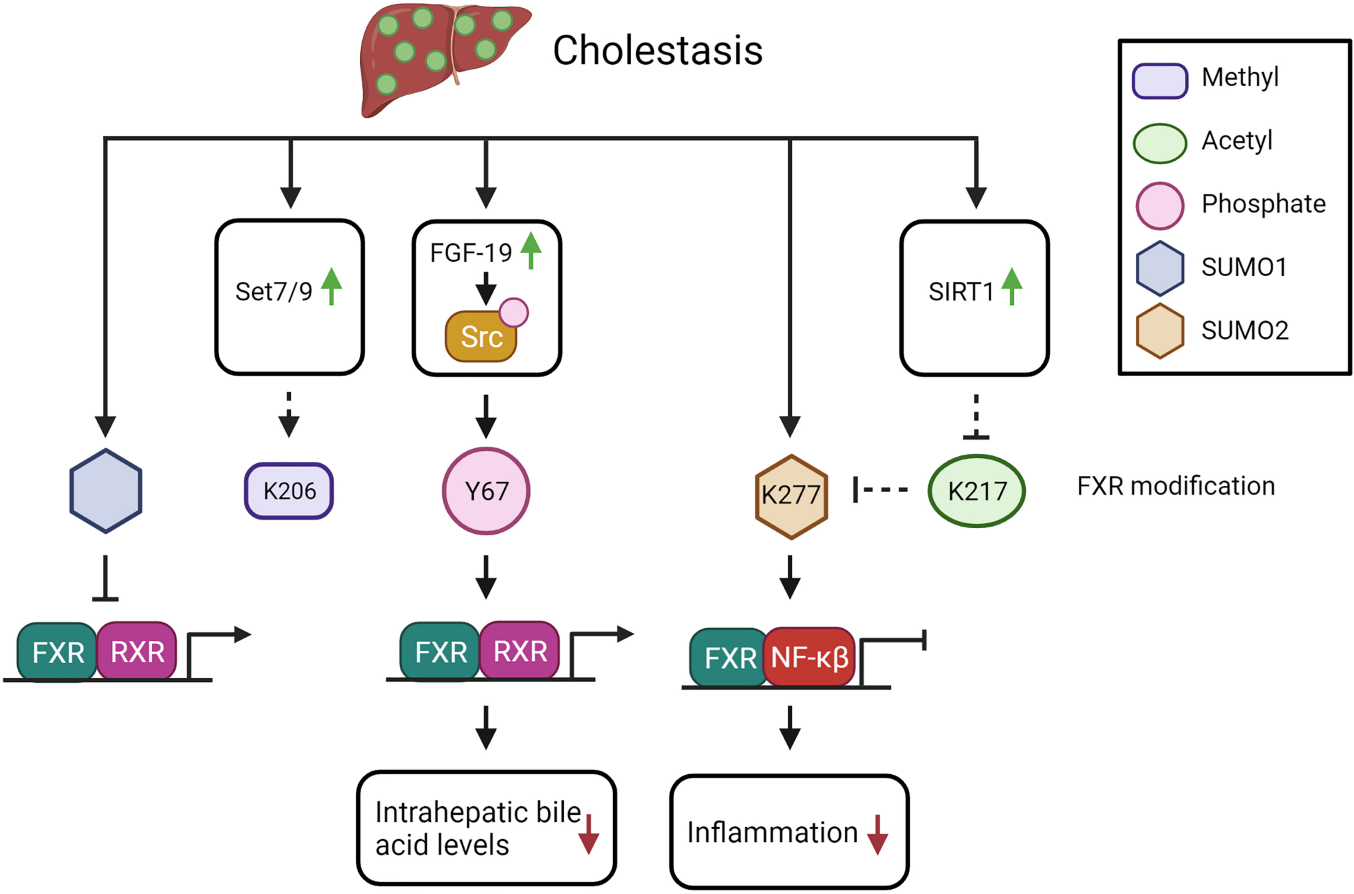
The potential impact of FXR PTMs in cholestasis (see text for explanation). Solid arrows indicate demonstrated relations or interactions, dashed lines indicate predictions inferred from the described molecular data.

FXR-Y67 phosphorylation is demonstrated to be increased in intrahepatic cholestasis induced by treatment with α-naphthyl isothiocyanate (ANIT). ANIT-mediated cholestasis leads to biliary epithelial cell injury (47). As described above, FXR-Y67 phosphorylation is regulated by FGF-19-mediated induction of Src (see **Figure 2**). Interestingly, FGF-19 expression is increased in cirrhotic patients, therefore we speculate that the increased FGF-19 could lead to elevation of FXR-Y67 phosphorylation in cirrhosis patients (88). In FXR-Y67F mutant mice, the cholestasis induced by acute bile acid feeding was more severe compared to control mice. FXR-Y67F mice had increased bile acid concentrations in the gallbladder and serum, as well as elevated liver toxicity markers (47). Expression of BSEP and SHP was reduced compared to WT mice (47). Thus, phosphorylation of FXR-Y67 in cholestasis is predicted to reduce intrahepatic bile acid levels *via* BSEP activation, in line with a protective effect during cholestasis.

Whether methylation of FXR-K206 is affected during cholestasis is not yet elucidated as far as we know. However, Set7/9 expression is increased after BDL, which may induce FXR methylation status (89). Methylation of FXR would increase BSEP expression, so potentially be protective in cholestasis by reducing the intrahepatic bile acid levels.

In livers of chronic cholestatic liver disease patients (primary biliary cirrhosis and primary sclerosing cholangitis) and in BDL mice, SIRT1 expression is upregulated, suggesting that FXR is less acetylated during cholestasis (90). The increase in SIRT1 expression could be recapitulated *in vitro* by treatment with high concentrations of bile acids in THLE-2 cells (liver epithelial cells of human origin). Interestingly, while BDL in WT mice induced the expressing of FXR, BDL in hepatocyte depleted SIRT1 mice (SIRT1^hep-/-^), resulted in a lower FXR protein expression correlating with lower SHP expression (90). Interestingly, the authors observed reduced FXR acetylation after BDL in both WT and in SIRT1^hep-/-^ mice (90). This demonstrates that FXR acetylation is reduced during cholestasis due to increased SIRT1 expression (see **Figure 2**), which leads to increased FXR target gene expression, as is predicted to ameliorate the cholestasic phenotype.

In progressive familiar intrahepatic cholestasis (PFIC) type 1, the cholestasis is caused by ATP8B1 deficiency. This protein flips PS from the outside to the inside of the cell where it activates PKCζ. As described above, PKCζ phosphorylates FXR-T442, which enhances the expression of FXR target gene BSEP, leading to increased bile acid export (55). As a result of the ATP8B1 deficiency, PFIC1 patients would have less FXR-T442 phosphorylation with a concomitant decrease in FXR function, contributing to the cholestatic phenotype. Taken together, the balance between the different PTMs will likely determine the end effect on FXR protein function in cholestasis.

### Obesity-Related Disorders

Obesity, as defined by having a Body Mass Index higher than 30, is caused by an imbalance between energy intake (food) and energy expenditure (basal metabolic functions and physical activity), leading to increased fat storage in adipose tissue and in major organs including the liver and the heart. This will eventually result in inflammation, insulin resistance and diabetes mellitus type 2 (DM2) and/or NAFLD (30, 31). NAFLD ranges from simple steatosis, to nonalcoholic steatohepatitis (NASH), which is characterized by steatosis, inflammation, and fibrosis (28, 29).

**Figure 3:**
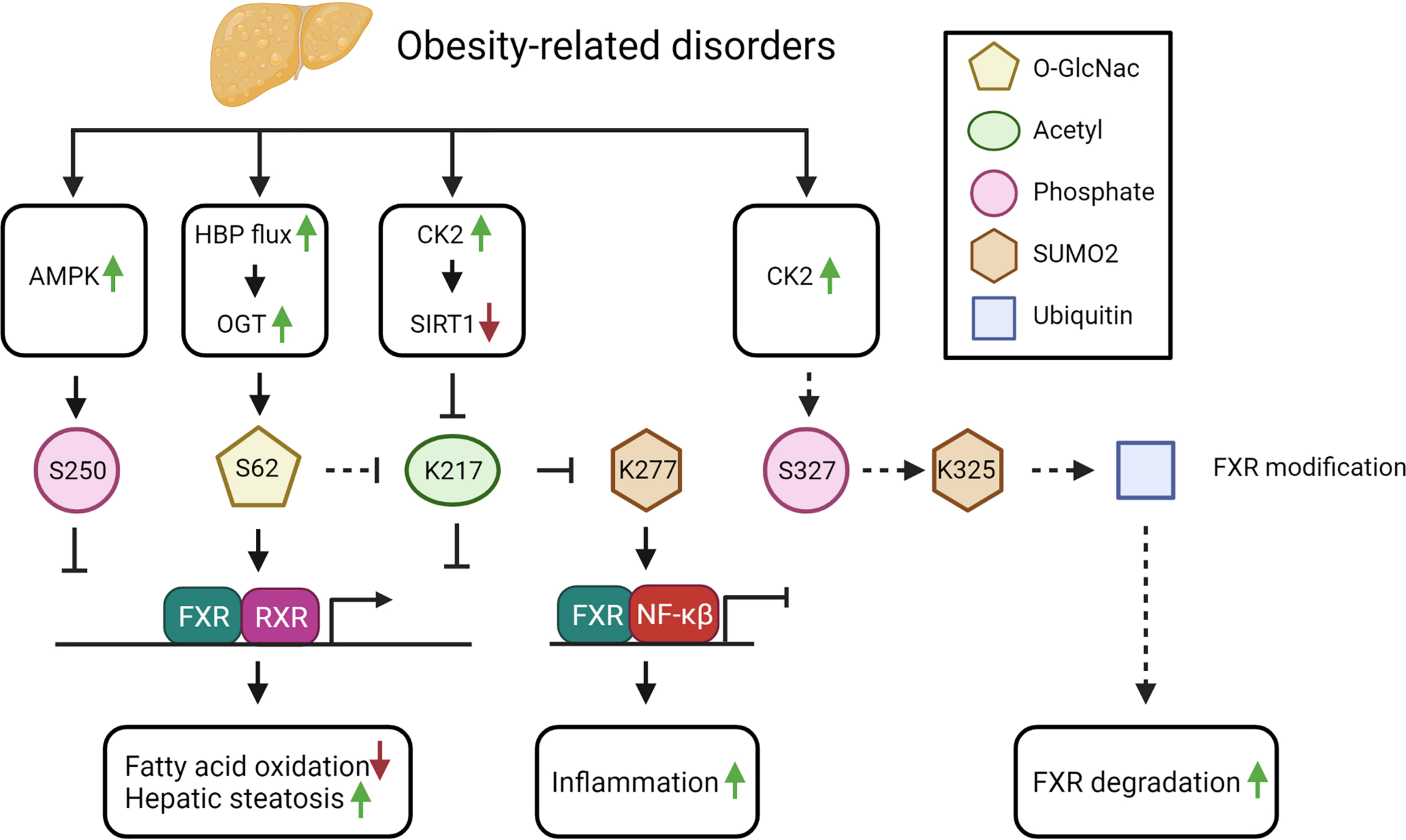
The potential impact of FXR PTMs in obesity-related disorders (see text for explanation). Solid arrows indicate demonstrated relations and/or interactions, dashed lines indicate predictions inferred from the described molecular data.

FXR-K277 SUMOylation is reduced in diet-induced obese mice compared to lean mice. Interestingly, the SUMOylated FXR gradually decreased over time and was nearly undetectable after 12 weeks of high fat diet (42). Because this modification has been described to reduce NF-κB mediated transcription, this would result in a potential increase in NF-κB mediated transcription and the inflammatory response in obesity (42) (see **Figure 3**).

Furthermore, protein acetylation in general, and FXR acetylation more specifically, is persistently elevated in diet-induced obese mice, seemingly as a consequence of the excess in acetyl-CoA precursors derived from unused energy substrates (41). An increase in FXR acetylation is predicted to lead to elevated liver cytokines and increased weight gain, as demonstrated by acetyl-mimetic FXR mutant mice, in which both features were observed under normal feeding conditions. In line with this, expressing an acetyl-defective mutant in obese mice, resulted in reduced hepatic inflammation, decreased lipid levels and increased glucose tolerance (42). Thus, hyperacetylation of FXR in obesity will contribute to hepatic inflammation and to dysregulated metabolism (see **Figure 3**).

Next to the excess in substrates for acetylation, an imbalance between the SIRT1 and P300 activity may furthermore impact FXR hyperacetylation. In obesity, SIRT1 phosphorylation is increased due to elevation of CK2 levels, which inhibits SIRT1 (91). Hence, inhibition of SIRT1 will result in increased FXR acetylation (41). In NAFLD patients, the levels of phosphorylated SIRT1 and CK2 correlate with disease severity (91). The observed increase in phosphorylated SIRT1 could be linked to a reduced SIRT1 mRNA expression in NASH patients. These studies suggest that during obesity-related disorders, the modification of FXR switches from SUMOylation (FXR-K277) towards acetylation (FXR-K217). Indeed, loss of SUMOylation could be linked to an increase of FXR acetylation in obese mice (42). The reduction of SUMO-modified FXR was shown to be caused by the hyperacetylation of FXR-K217 (see **Figure 3**), which prevents binding of the SUMO E3 ligase PIASγ to FXR (42).

The increase in CK2 expression in obesity could also lead to an elevation in FXR-S327 phosphorylation, which in response would increase FXR-S327-phosphorylation-dependent SUMOylation of FXR-K325. The latter would potentially lead to increased FXR ubiquitination and degradation, thereby reducing FXR protein expression. In line with this, FXR expression is reduced in rodent models of both diabetes and obesity (92, 93). On the contrary however, increased FXR acetylation may result in a more stable protein due to increased FXR-RXRα interaction. Unfortunately, these studies did not investigate FXR SUMOylation or ubiquitination, nor FXR protein expression, so we cannot verify these hypotheses. Because of the reduced FXR expression, it would be interesting to investigate FXR ubiquitination and its binding to RNF4 in NAFLD.

In diabetes type 2 (DM2), the increased glucose concentration increases the flux through the hexamine biosynthetic pathway (HBP) of which the major end product, UDP-N-acetylglucosamine (UDP-GlcNAc), is the essential substrate of OGT, the enzyme required for O-GlcNAcylation of proteins (94). As expected, an increased flux though the HBP combined with the increased UDP-GlcNAc concentration, leads to increased protein O-GlcNAcylation in individuals with DM2 (95). Moreover, *in vitro*, the high glucose concentration could be directly linked to the increased O-GlcNAcylation, including FXR O-GlcNAcylation (45). The effect of increased O-GlcNAcylation of FXR resulted in increased FXR target gene expression as demonstrated in BSEP (96) (see **Figure 3**). In case of DM2, this potentially could lead to an increase in the FXR target genes Kruppel Like Factor 11 (KLF11) and SHP inducing an increase in insulin sensitivity and a reduction in plasma glucose levels respectively (97).

Thus, in obesity-related disorders, multiple substrates and PTM-inducing enzymes are altered in abundance and expression, leading to changes in FXR PTMs (see **Figure 3**). Further studies should address how changing the PTM balance would affect obesity and related disorders.

## Conclusions and Future Research

FXR plays a pivotal role in the regulation of bile acid, glucose, fat and amino acid metabolism. Pharmacological activation of FXR provides protection in certain cholestasic and metabolic conditions in mice and humans. This review provides an overview of the complex regulation of FXR at the post-translational level and their effects on FXR (target gene) expression and function. In addition, we elaborated on the potential effects of the several PTMs in different disease states. However, the individual and combined contribution of each of these PTMs under various disease conditions remains largely unclear. One could imagine that these regulatory systems complement each other to ensure effective regulation of bile acid, glucose, fat and amino acid metabolism. Alternatively, specific modifications can also block the addition of other modifications. Therefore, several blind spots still exist in our knowledge about the effect of the PTMs on FXR function. The crystal structure of FXR in various conformations has been elucidated. Insight into the 3D structure and conformational changes upon individual PTMs would be highly valuable to understand the individual contribution and additive effect of the PTMs on FXR expression and function. In addition, insight into the quaternary structure of FXR, involving subunit interaction and interaction with other nuclear receptors and/or NF-κB, or adaptor proteins would be valuable in this regard. Finally, it seems that there is selectivity for the recruitment of PTM-mediating enzymes to different DNA loci in the genome, since some FXR target genes are affected by a particular modification, while others are not. Therefore, future research should address global DNA binding and transcriptome effects of different PTMs. These additional insights in specific PTMs will help us in rationalizing whether a particular PTM should be targeted in cholestasis and obesity-related disorders.

## Author Contributions

MA and SV, Conceptualization, Writing – original draft, Writing – review and editing, Visualization. SM, Conceptualization, Writing – original draft, Writing – review and editing, Funding. All authors contributed to the article and approved the submitted version.

## Funding

This work was financially supported by the Netherlands Organisation for Health Research and Development (ZonMW; VICI grant, no: 09150181910029 and Aspasia grant, no: 015.015.013).

## Conflict of Interest

The authors declare that the research was conducted in the absence of any commercial or financial relationships that could be construed as a potential conflict of interest.

## Publisher’s Note

All claims expressed in this article are solely those of the authors and do not necessarily represent those of their affiliated organizations, or those of the publisher, the editors and the reviewers. Any product that may be evaluated in this article, or claim that may be made by its manufacturer, is not guaranteed or endorsed by the publisher.

## Abbreviations

AF, activation function domain; AMPK, 5’ AMP-activated protein kinase; ANIT, α-naphtyl isothiocynate; ATP8B1, ATPase phospholipid transporting 8B1; BDL, bile duct ligation; BSEP, bile salt export pump; CA, cholic acid; CDCA, chenodeoxycholic acid; ChIP, chromatin immune-precipitation; CK2, casein kinase 2; DBD, DNA binding domain; DCA, deoxycholic acid; DM2, diabetes mellitus type 2; ER-2, everted hexamer repeat sequence spaced by 2 nucleotides; FGF-15/19, fibroblast growth factor 15/19; FGFR4, fibroblast growth factor receptor 4; FXR, farnesoid X receptor; FXRE, FXR response element; HBP, hexamine biosynthetic pathway; IBABP, ileal bile acid binding protein; IR-1, inverted hexamer repeat sequence spaced by 1 nucleotide; KLF11, kruppel like factor 11; LBD, ligand binding domain; LCA, lithocholic acid; LPS, lipopolysaccharide; MCA, muricholic acid; MGEA5, meningioma expressed antigen 5; NAFLD, non-alcoholic fatty liver disease; NASH, non-alcoholic steatohepatitis; NF-κB, Nuclear factor kappa beta; NR1H, nuclear Receptor Subfamily group H; OCA, obeticholic acid; OGT, O-GlcNAc transferase; Ostα, organic solute transporter complex alpha; Ostβ, organic solute transporter complex beta; PFIC, progressive familial intrahepatic cholestasis; PIAS, protein inhibitor of activated STAT; PKC, protein kinase C; PS, phosphatidylserine; PTM, post-translational modification; RNF4, RING-domain-containing-ubiquitin E3 ligase; RXR, retinoid X receptor; SHP, small heterodimeric partner; SHP-2, Src homology region 2 domain-containing phosphatase-2; SIRT1, sirtuin 1; Src, proto-oncogene tyrosine-protein kinase Src; SUMO, small ubiqituin-like modifier; TCA, taurocholic acid; UBE2I, ubiquitin conjugating enzyme E2 I; UDCA, ursodeoxycholic acid; UDP-GlcNAc, UDP-N-acetylglucosamine; WT, wild type.
